# 2-(Dibromo­meth­yl)benzoic acid

**DOI:** 10.1107/S1600536811052858

**Published:** 2011-12-14

**Authors:** Hong-Yi Lin, Sin-Kai Fang, Kew-Yu Chen

**Affiliations:** a Department of Chemical Engineering, Feng Chia University, 40724 Taichung, Taiwan

## Abstract

In the crystal structure of the title compound, C_8_H_6_Br_2_O_2_, the carboxyl groups are involved in pairs of O—H⋯O hydrogen bonds, which link the mol­ecules into inversion dimers.

## Related literature

For the preparation of the title compound, see: Eliel & Rivard (1952 ▶). For its applications, see: Dey & Mal (2005 ▶). For graph-set theory, see: Bernstein *et al.* (1995 ▶).
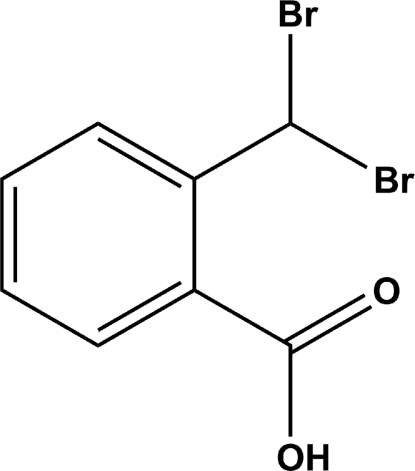


## Experimental

### 

#### Crystal data

C_8_H_6_Br_2_O_2_
*M* *_r_* = 293.95
Monoclinic, 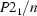

*a* = 4.9988 (6) Å
*b* = 25.617 (3) Å
*c* = 7.1844 (8) Å
β = 97.709 (10)°
*V* = 911.68 (18) Å^3^
*Z* = 4
Mo *K*α radiation
μ = 8.85 mm^−1^
*T* = 297 K
0.74 × 0.36 × 0.25 mm

#### Data collection

Bruker SMART CCD area-detector diffractometer
Absorption correction: multi-scan (*SADABS*; Bruker, 2001 ▶) *T*_min_ = 0.251, *T*_max_ = 1.000
7515 measured reflections
2210 independent reflections
1221 reflections with *I* > 2σ(*I*)
*R*_int_ = 0.088

#### Refinement

*R*[*F* ^2^ > 2σ(*F* ^2^)] = 0.091
*wR*(*F* ^2^) = 0.227
*S* = 1.13
2210 reflections
109 parameters
H-atom parameters constrained
Δρ_max_ = 0.85 e Å^−3^
Δρ_min_ = −0.93 e Å^−3^

### 

Data collection: *SMART* (Bruker, 2001 ▶); cell refinement: *SAINT* (Bruker, 2001 ▶); data reduction: *SAINT*; program(s) used to solve structure: *SHELXS97* (Sheldrick, 2008 ▶); program(s) used to refine structure: *SHELXL97* (Sheldrick, 2008 ▶); molecular graphics: *ORTEP-3 for Windows* (Farrugia, 1997 ▶); software used to prepare material for publication: *WinGX* (Farrugia, 1999 ▶).

## Supplementary Material

Crystal structure: contains datablock(s) I, global. DOI: 10.1107/S1600536811052858/lx2208sup1.cif

Structure factors: contains datablock(s) I. DOI: 10.1107/S1600536811052858/lx2208Isup2.hkl

Supplementary material file. DOI: 10.1107/S1600536811052858/lx2208Isup3.cml

Additional supplementary materials:  crystallographic information; 3D view; checkCIF report

## Figures and Tables

**Table 1:** Hydrogen-bond geometry (Å, °)

*D*—H⋯*A*	*D*—H	H⋯*A*	*D*⋯*A*	*D*—H⋯*A*
O2—H2*A*⋯O1^i^	0.82	1.82	2.641 (11)	176

Symmetry code: (i) 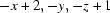.

**Fig. 1.**
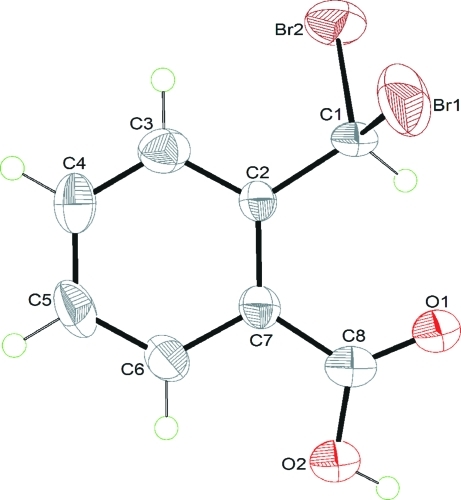
The molecular structure of the title compound with the atom numbering scheme. Displacement ellipsoids are drawn at the 50% probability level. H atoms are presented as small spheres of arbitrary radius.

**Fig. 2.**
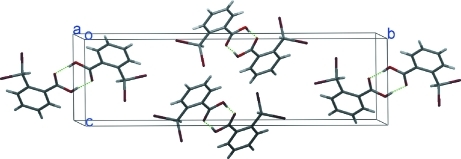
A view of the O–H···O hydrogen bonds (dotted lines) in the crystal structure of the title compound.

## supplementary crystallographic information

### Comment

The title compound is a useful reagent to prepare phthalaldehydic acid (Eliel & Rivard, 1952). In addition, it has been prepared as a potential precursor to an antitumour agent, BE-23254. (Dey & Mal, 2005). The structure of the title compound is shown in Fig. 1. In the crystal structure (Fig. 2), inversion-related molecules are linked by pairs of O–H···O hydrogen bonds, forming a cyclic dimers with *R*_2_^2^(8) graph-set motif (Table 1) (Bernstein *et al.*, 1995). The intramolecular C–H···O hydrogen bond (Table 1) generates an S(6) ring motif .

### Experimental

The title compound was synthesized according to the literature (Eliel & Rivard, 1952). Single crystals suitable for X-ray diffraction were prepared by slow evaporation of a solution of the title compound in chloroform at room temperature for six weeks.

### Refinement

The C bound H atoms positioned geometrically (C–H = 0.93-0.98 Å) and allowed to ride on their parent atoms, with *U*_iso_(H) = 1.2*U*_eq_(C)]. The O bound H atoms positioned geometrically (O–H = 0.82 Å) and allowed to ride on their parent atoms, with *U*_iso_(H) =1.5*U*_eq_(O)].

### Crystal data

**Table:**

C_8_H_6_Br_2_O_2_	*F*(000) = 560
*M**_r_* = 293.95	*D*_x_ = 2.142 Mg m^−^^3^
Monoclinic, *P*2_1_/*n*	Mo *K*α radiation, λ = 0.71073 Å
Hall symbol: -P 2yn	Cell parameters from 2762 reflections
*a* = 4.9988 (6) Å	θ = 2.9–29.2°
*b* = 25.617 (3) Å	µ = 8.85 mm^−^^1^
*c* = 7.1844 (8) Å	*T* = 297 K
β = 97.709 (10)°	Parallelepiped, colorless
*V* = 911.68 (18) Å^3^	0.74 × 0.36 × 0.25 mm
*Z* = 4	

### Data collection

**Table:**

Bruker SMART CCD area-detector diffractometer	2210 independent reflections
Radiation source: fine-focus sealed tube	1221 reflections with *I* > 2σ(*I*)
graphite	*R*_int_ = 0.088
ω scans	θ_max_ = 29.3°, θ_min_ = 3.0°
Absorption correction: multi-scan (*SADABS*; Bruker, 2001)	*h* = −6→6
*T*_min_ = 0.251, *T*_max_ = 1.000	*k* = −34→34
7515 measured reflections	*l* = −9→9

### Refinement

**Table:**

Refinement on *F*^2^	Primary atom site location: structure-invariant direct methods
Least-squares matrix: full	Secondary atom site location: difference Fourier map
*R*[*F*^2^ > 2σ(*F*^2^)] = 0.091	Hydrogen site location: difference Fourier map
*wR*(*F*^2^) = 0.227	H-atom parameters constrained
*S* = 1.13	*w* = 1/[σ^2^(*F*_o_^2^) + (0.0876*P*)^2^ + 4.8672*P*] where *P* = (*F*_o_^2^ + 2*F*_c_^2^)/3
2210 reflections	(Δ/σ)_max_ = 0.001
109 parameters	Δρ_max_ = 0.85 e Å^−^^3^
0 restraints	Δρ_min_ = −0.93 e Å^−^^3^

### Special details

**Table:**

Geometry. All e.s.d.'s (except the e.s.d. in the dihedral angle between two l.s. planes) are estimated using the full covariance matrix. The cell e.s.d.'s are taken into account individually in the estimation of e.s.d.'s in distances, angles and torsion angles; correlations between e.s.d.'s in cell parameters are only used when they are defined by crystal symmetry. An approximate (isotropic) treatment of cell e.s.d.'s is used for estimating e.s.d.'s involving l.s. planes.
Refinement. Refinement of *F*^2^ against ALL reflections. The weighted *R*-factor *wR* and goodness of fit *S* are based on *F*^2^, conventional *R*-factors *R* are based on *F*, with *F* set to zero for negative *F*^2^. The threshold expression of *F*^2^ > σ(*F*^2^) is used only for calculating *R*-factors(gt) *etc*. and is not relevant to the choice of reflections for refinement. *R*-factors based on *F*^2^ are statistically about twice as large as those based on *F*, and *R*- factors based on ALL data will be even larger.

### Fractional atomic coordinates and isotropic or equivalent isotropic displacement parameters (Å^2^)

**Table:**

	*x*	*y*	*z*	*U*_iso_*/*U*_eq_	
Br1	0.4317 (3)	0.14770 (6)	0.73655 (16)	0.0676 (5)	
Br2	0.6793 (3)	0.22661 (4)	0.4627 (2)	0.0700 (5)	
O1	0.8610 (16)	0.0565 (3)	0.5279 (11)	0.055 (2)	
O2	0.7203 (17)	−0.0025 (3)	0.3117 (11)	0.057 (2)	
H2A	0.8523	−0.0181	0.3649	0.086*	
C1	0.613 (2)	0.1540 (4)	0.5132 (14)	0.038 (2)	
H1A	0.7859	0.1356	0.5355	0.046*	
C2	0.4355 (19)	0.1276 (3)	0.3520 (12)	0.032 (2)	
C3	0.216 (2)	0.1550 (4)	0.2570 (14)	0.043 (2)	
H3A	0.1840	0.1890	0.2926	0.052*	
C4	0.050 (2)	0.1329 (4)	0.1143 (14)	0.045 (3)	
H4A	−0.0943	0.1519	0.0528	0.054*	
C5	0.093 (2)	0.0821 (4)	0.0596 (14)	0.050 (3)	
H5A	−0.0223	0.0667	−0.0375	0.060*	
C6	0.307 (2)	0.0547 (4)	0.1501 (14)	0.041 (2)	
H6A	0.3367	0.0207	0.1115	0.049*	
C7	0.484 (2)	0.0759 (4)	0.2988 (12)	0.033 (2)	
C8	0.702 (2)	0.0430 (4)	0.3896 (14)	0.037 (2)	

### Atomic displacement parameters (Å^2^)

**Table:**

	*U*^11^	*U*^22^	*U*^33^	*U*^12^	*U*^13^	*U*^23^
Br1	0.0750 (9)	0.0939 (10)	0.0320 (6)	0.0025 (8)	0.0000 (5)	−0.0081 (6)
Br2	0.0771 (10)	0.0335 (6)	0.0951 (11)	−0.0055 (6)	−0.0039 (8)	−0.0100 (6)
O1	0.060 (5)	0.039 (4)	0.062 (5)	0.013 (4)	−0.013 (4)	−0.011 (4)
O2	0.065 (5)	0.036 (4)	0.065 (5)	0.011 (4)	−0.011 (4)	−0.011 (4)
C1	0.043 (6)	0.028 (5)	0.042 (6)	0.007 (4)	0.003 (5)	−0.010 (4)
C2	0.040 (5)	0.030 (4)	0.024 (4)	0.002 (4)	−0.002 (4)	0.000 (4)
C3	0.056 (7)	0.037 (5)	0.037 (6)	0.004 (5)	0.010 (5)	0.000 (4)
C4	0.039 (6)	0.062 (7)	0.032 (5)	−0.003 (5)	0.000 (4)	0.009 (5)
C5	0.057 (7)	0.060 (7)	0.029 (5)	−0.013 (6)	−0.009 (5)	−0.004 (5)
C6	0.034 (5)	0.045 (6)	0.043 (6)	−0.001 (5)	0.002 (4)	−0.013 (5)
C7	0.044 (6)	0.034 (5)	0.022 (4)	0.000 (4)	0.004 (4)	0.001 (4)
C8	0.042 (6)	0.031 (5)	0.040 (6)	−0.002 (4)	0.016 (5)	−0.001 (4)

### Geometric parameters (Å, °)

**Table:**

Br1—C1	1.951 (10)	C3—C4	1.354 (14)
Br2—C1	1.932 (10)	C3—H3A	0.9300
O1—C8	1.235 (12)	C4—C5	1.386 (15)
O2—C8	1.302 (11)	C4—H4A	0.9300
O2—H2A	0.8200	C5—C6	1.370 (15)
C1—C2	1.519 (13)	C5—H5A	0.9300
C1—H1A	0.9800	C6—C7	1.402 (13)
C2—C3	1.399 (14)	C6—H6A	0.9300
C2—C7	1.409 (12)	C7—C8	1.461 (14)
			
C8—O2—H2A	109.5	C3—C4—H4A	119.9
C2—C1—Br2	112.5 (7)	C5—C4—H4A	119.9
C2—C1—Br1	107.6 (7)	C6—C5—C4	119.3 (9)
Br2—C1—Br1	110.2 (4)	C6—C5—H5A	120.4
C2—C1—H1A	108.8	C4—C5—H5A	120.4
Br2—C1—H1A	108.8	C5—C6—C7	122.4 (9)
Br1—C1—H1A	108.8	C5—C6—H6A	118.8
C3—C2—C7	119.4 (9)	C7—C6—H6A	118.8
C3—C2—C1	119.2 (8)	C2—C7—C6	117.3 (9)
C7—C2—C1	121.4 (8)	C2—C7—C8	124.5 (9)
C4—C3—C2	121.5 (10)	C6—C7—C8	118.2 (9)
C4—C3—H3A	119.2	O1—C8—O2	121.5 (9)
C2—C3—H3A	119.2	O1—C8—C7	123.9 (9)
C3—C4—C5	120.2 (10)	O2—C8—C7	114.6 (9)
			
Br2—C1—C2—C3	−40.2 (11)	C1—C2—C7—C6	179.1 (9)
Br1—C1—C2—C3	81.4 (9)	C3—C2—C7—C8	−178.5 (9)
Br2—C1—C2—C7	141.2 (8)	C1—C2—C7—C8	0.1 (15)
Br1—C1—C2—C7	−97.3 (9)	C5—C6—C7—C2	−0.8 (15)
C7—C2—C3—C4	−0.2 (15)	C5—C6—C7—C8	178.3 (10)
C1—C2—C3—C4	−178.9 (9)	C2—C7—C8—O1	2.9 (16)
C2—C3—C4—C5	0.4 (16)	C6—C7—C8—O1	−176.1 (10)
C3—C4—C5—C6	−0.7 (16)	C2—C7—C8—O2	−176.3 (9)
C4—C5—C6—C7	0.9 (17)	C6—C7—C8—O2	4.7 (13)
C3—C2—C7—C6	0.4 (14)		

### Hydrogen-bond geometry (Å, °)

**Table:**

*D*—H···*A*	*D*—H	H···*A*	*D*···*A*	*D*—H···*A*
O2—H2A···O1^i^	0.82	1.82	2.641 (11)	176
C1—H1A···O1	0.98	2.06	2.784 (13)	129

Symmetry codes: (i) −*x*+2, −*y*, −*z*+1.
